# Genome Sequences of Five Type Strain Members of the Archaeal Family *Sulfolobaceae*, Acidianus ambivalens, Acidianus infernus, Stygiolobus azoricus, Sulfuracidifex metallicus, and Sulfurisphaera ohwakuensis

**DOI:** 10.1128/MRA.01490-19

**Published:** 2020-03-12

**Authors:** James A. Counts, Nicholas P. Vitko, Robert M. Kelly

**Affiliations:** a Department of Chemical and Biomolecular Engineering, North Carolina State University, Raleigh, North Carolina, USA; Portland State University

## Abstract

Presented are five genomes from the polyextremophilic (optimal temperature of >65°C and optimal pH of <3.5) archaeal family *Sulfolobaceae*, greatly expanding order-wide genomic diversity. Included are the only obligate anaerobic species, several facultative sulfur utilizers, two metal mobilizers, one facultative chemolithoautotroph with robust metabolic versatility, and some of the most thermophilic thermoacidophiles reported to date.

## ANNOUNCEMENT

Extremely thermoacidophilic *Sulfolobales* (*Archaea*, *Crenarchaeota*, *Thermoprotei*, *Sulfolobales*, *Sulfolobaceae*) are ubiquitous in highly acidic geothermal environments around the world. These organisms subsist in inorganic component-rich (sulfur-rich, metal-rich, and organic carbon-limited) environments, ranging from calderas to mud pits to sulfur pools, and act as microbial transformers of inorganic, energetic materials resulting from subsurface terrestrial activities. The genomes presented here are for the strains Acidianus ambivalens DSM 3772 (1), Acidianus infernus DSM 3191 (2), Stygiolobus azoricus DSM 6296 (3), Sulfuracidifex metallicus DSM 6482 (4), and Sulfurisphaera ohwakuensis DSM 12421 (5), which have diverse metabolic capabilities and are globally distributed (Table 1). This work represents a second stage in sequencing that complements previous work focused on metal biooxidizers of the genera *Metallosphaera* and *Acidianus* (6). Additionally, this research reports three previously unsequenced type strains and provides more complete information for two recent genome-sequencing projects associated with the other two type strains.

**TABLE 1:** Collection, sequencing, assembly, and characteristics of sequenced species

	Data for:				
Parameter	Acidianus ambivalens	Acidianus infernus	Stygiolobus azoricus	Sulfuracidifex metallicus	Sulfurisphaera ohwakuensis
Collection identification no.	DSM 3772^T^, JCM9191^T^	DSM 3191^T^, JCM8955^T^	DSM 6296^T^, JCM9021^T^	DSM 6482^T^, JCM9184^T^	DSM 12421^T^, JCM9065^T^
Isolation site	Leirhnjúkur, Mývatn, Iceland	Pisciarelli Solfatara, Naples, Italy	São Miguel Island, Azores	Krafla, Mývatn, Iceland	Hot spring in Ohwaku Valley, Hakone, Japan
Metabolism	Chemolithoautotrophic	Chemolithoautotrophic	Chemolithoautotrophic	Chemolithoautotrophic	Chemolithoautotrophic
Oxygen metabolism	Facultative	Facultative	Anaerobic	Aerobic	Facultative
Optimal temp (°C)	80	90	80	65	85
Optimal pH	2.0	2.0	2.5	2.0	2.0
No. of long reads (coverage)	299,403 (661×)^*a*^	104,250 (438×)^*a*^^,^^*b*^	185,364 (482×)^*a*^	62,361 (308×)^*b*^^,^^*c*^	151,460 (362×)^*a*^
No. of short reads (coverage)	3,961,682 (260×)^*d*^	4,171,032 (469×)^*e*^	4,830,704 (605×)^*e*^	5,477,710 (620×)^*e*^	5,400,166 (481×)^*e*^
Genome size (bp)	2,252,027^*f*^	2,220,671	1,987,069^*f*^	2,199,731	2,803,915^*f*^
No. of contigs (size of largest contig [bp])		4 (2,184,866)		5 (1,839,470)	
G+C content (mol%)	34.2	34.4	37.6	38.6	32.7
BioProject accession no.	PRJNA488459	PRJNA488459	PRJNA488459	PRJNA463410	PRJNA488459
BioSample accession no.	SAMN09933089	SAMN09933090	SAMN09933091	SAMN09933021	SAMN09933092
Genome accession no.	CP045482	WFIY00000000	CP045483	WGGD00000000	CP045484
Read accession no.					
PacBio	SRX7690445	SRX7690447	SRX7690449	SRR11038957	SRX7690451
Illumina	SRX7690446	SRX7690448	SRX7690450	SRR11038956	SRX7690452

a PacBio Sequel (3-kb selection).

b Trimmomatic filtered or long-read filtered (>5,000 bp for *A. infernus* and >8,000 bp for *S. metallicus*).

c PacBio RS II (15-kb selection).

d Illumina NextSeq 500 (single-end 150-bp reads).

e Illumina MiSeq (paired-end 250-bp reads).

f Closed.

All of the species presented here were obtained from isolates deposited at the Deutsche Sammlung von Mikroorganismen und Zellkulturen GmbH (DSMZ). All of the cultures were grown as recommended by the DSMZ; furthermore, the *Acidianus* spp. and S. azoricus were cultured anaerobically with a hydrogen/carbon dioxide (20:80) headspace. Organic solvent-based DNA extraction was performed with phenol, chloroform, and isoamyl alcohol, as well as an isopropanol precipitation, similar to the method described by Geslin et al. (7).

Single-molecule real-time (SMRT) sequencing was performed with either a SMRT cell with the RS II system or multiplexing (Barcoded Adapter kit 8A) in a SMRT cell with the Sequel system (Pacific Biosciences, Menlo Park, CA, USA). Both library configurations were size selected with BluePippin (15 or 3 kb) and prepared with a SMRTbell template preparation kit 1.0-SPv3. Additional short-read data were prepared as either single-end 150-bp reads or paired-end 250-bp reads using the TruSeq Nano DNA v2 library preparation kit, on either a MiSeq or NextSeq 500 sequencer (Illumina, San Diego, CA, USA). Assemblies were performed with a recent repeat graph assembler designed for long error-prone reads, Flye (v2.4.2), with default parameters and a 2.5-Mb genome size estimate (8), using raw consensus long-read data (Table 1). Following the initial assembly, short-read data were mapped to all of the constructs using Bowtie 2 (v2.3.2) (9), with medium sensitivity, in order to correct for sequencing errors from long-read data. These short reads were curated with Trimmomatic v0.38 (10), with the criteria of phred33 scores greater than 30 for head and tail and 28 within a sliding window of 4 bases and with the removal of reads of less than 145 bp (single-end reads) and 240 bp (paired-end reads). Finally, genomes were deposited in the NCBI genome database, where they were annotated via the PGAAP algorithm (11).

In summation, five genome sequences, representing diverse members of the family *Sulfolobaceae*, are presented. While the *S. metallicus* genome remains unclosed, the draft genome provided here greatly improves on a previous short-read assembly that produced 167 contigs (accession number BBBY01); the *A. ambivalens* genome (previously 67 contigs) (12) is now closed. These data provide new information to assist researchers in fields ranging from fundamental microbiology and ecology to biotechnology and metabolic engineering.

### Data availability.

All relevant BioProject, BioSample, and genome accession numbers are presented in Table 1.
